# National oncofertility registries around the globe: a pilot survey

**DOI:** 10.3389/fendo.2023.1148314

**Published:** 2023-05-08

**Authors:** Noelle Ozimek, Mahmoud Salama, Teresa K. Woodruff

**Affiliations:** ^1^ Harvard Medical School, Boston, MA, United States; ^2^ Oncofertility Consortium, Michigan State University, East Lansing, MI, United States

**Keywords:** oncofertility, registry, database, fertility preservation, cancer

## Abstract

**Purpose:**

Oncofertility is an emerging discipline which aims to preserve fertility of young cancer patients. As fertility preservation services have become increasingly available to cancer patients in many countries around the globe, it is crucial to establish a foundation of collaborative reporting to continuously monitor and assess oncofertility practices. This survey study investigates the current global landscape of official national oncofertility registries, a vital tool which allows for surveillance of the field.

**Methods:**

An online pilot survey was conducted to give the opportunity to report official national oncofertility registries available in 2022. Survey questions covered the availability of official national registries for oncofertility as well as the official national registries for cancer and assisted reproductive technologies. Participation in the survey was voluntary, anonymous and for free.

**Results:**

According to our online pilot survey, responses were collected from 20 countries including Argentina, Australia, Brazil, Canada, Chile, China, Egypt, Germany, Greece, India, Japan, Kenya, Philippines, Romania, South Africa, Thailand, Tunisia, UK, USA & Uruguay. Only 3 out of the 20 surveyed countries have well-established official national oncofertility registries; and include Australia, Germany & Japan. The Australian official national oncofertility registry is part of Australasian Oncofertility Registry that also includes New Zealand. The German official national oncofertility registry is part of FertiPROTEKT Network Registry for German speaking countries that also includes Austria & Switzerland. The Japanese official national oncofertility registry includes Japan only and called Japan Oncofertility Registry (JOFR). A supplementary internet search confirmed the aforementioned results. Therefore, the final list of countries around the globe that have official national oncofertility registries includes Australia, Austria, Germany, Japan, New Zealand, and Switzerland. Some other countries such as the USA and Denmark are on their way to establish official national registries for oncofertility care.

**Conclusion:**

Although oncofertility services are expanding globally, very few countries have well-established official national oncofertility registries. By reviewing such a global landscape, we highlight the urgent need for having a well-established official national oncofertility registry in each country to monitor oncofertility services in a way that best serves patients.

## 1 Introduction

Advancements in medicine have led to an increase in the survival rates of pediatric and adolescent cancers over the past decades (1). While these patients are recovering and living longer, common cancer treatments such as alkylating chemotherapy and ionizing radiation are highly gonadotoxic, resulting in fertility loss and a low chance of genetic parenthood for many survivors. The risk of cancer therapy-induced gonadotoxicity and fertility loss depends on the type and dose of cancer therapy, type and stage of the disease, as well as the age of the patient and the status of reproductive functions at the time of treatment (2). Therefore, there is a growing need to develop novel techniques which allow for the preservation and protection of fertility for pediatric, adolescent, and young adult cancer survivors. First coined in 2006, “Oncofertility” is an interdisciplinary field that bridges oncology and reproductive medicine with the goal of preserving the reproductive future of cancer survivors (3). The increasing availability of new oncofertility services such as ovarian and testicular tissue cryopreservation, in addition to the traditional sperm, egg and embryo cryobanking has generated a need to monitor these services across institutions and clinics.

Registries crucially provide an unparalleled opportunity for the surveillance of medical practices at all levels- local, regional, national, multinational, and globally. When registries are thorough, and data is complete and accurate, the information contained has the potential to shed light on epidemiological trends, highlight areas for improvement, and inform public health stakeholders. In any field, well-established national registries create opportunities for the surveillance and comparison of treatments on a large scale, allowing for the evaluation of novel approaches, and the continued safety monitoring of traditional approaches across the country. Ultimately, the information contained within a well-established national registry has the potential to guide informed decision making for both patient and provider, improve patient experience and reduce the burden of disease (4, 5).

Some of the most well-established registries are in the field of oncology (5). Such registries hold information on cancer prevalence, subtypes and treatment efficacy which have guided standards of care (6). For example, a study on registry data reported a marked disparity in mortality from cancer between developed and developing countries, with 57% of new cases and 65% of deaths in 2012 occurring in developing countries, painting a global picture that would have otherwise been more difficult to obtain (7). CONCORD, an established global surveillance program for cancer survival trends across 71 countries collected data from over 37.5 million cases in a 15-year period, which was used to inform on the global status of cancer and guide healthcare policy, speaking to the clear benefit of medical registries (8–10).

Compared to cancer registries, the degree to which Assisted Reproductive Technologies (ART) registries are established is more varied. The European IVF monitoring consortium is one such registry used to track the success of multiple reproductive technologies performed at over 1000 institutions from 43 countries (11). Nearly annual reports on this registry have uncovered a trend towards marginal improvement in efficacy of these services, among other findings (12–15). The quantity of data collected in this registry increases the impact of its studies and further highlights the importance of ART registries.

While some ART registries may include specialized oncofertility services, there is a general lack of recording this data in many countries around the globe. The main purpose of this study is to investigate the global existence of national oncofertility registries in relation to national cancer and ART registries.

## 2 Methods

### 2.1 Data collection

**Figure 1:**
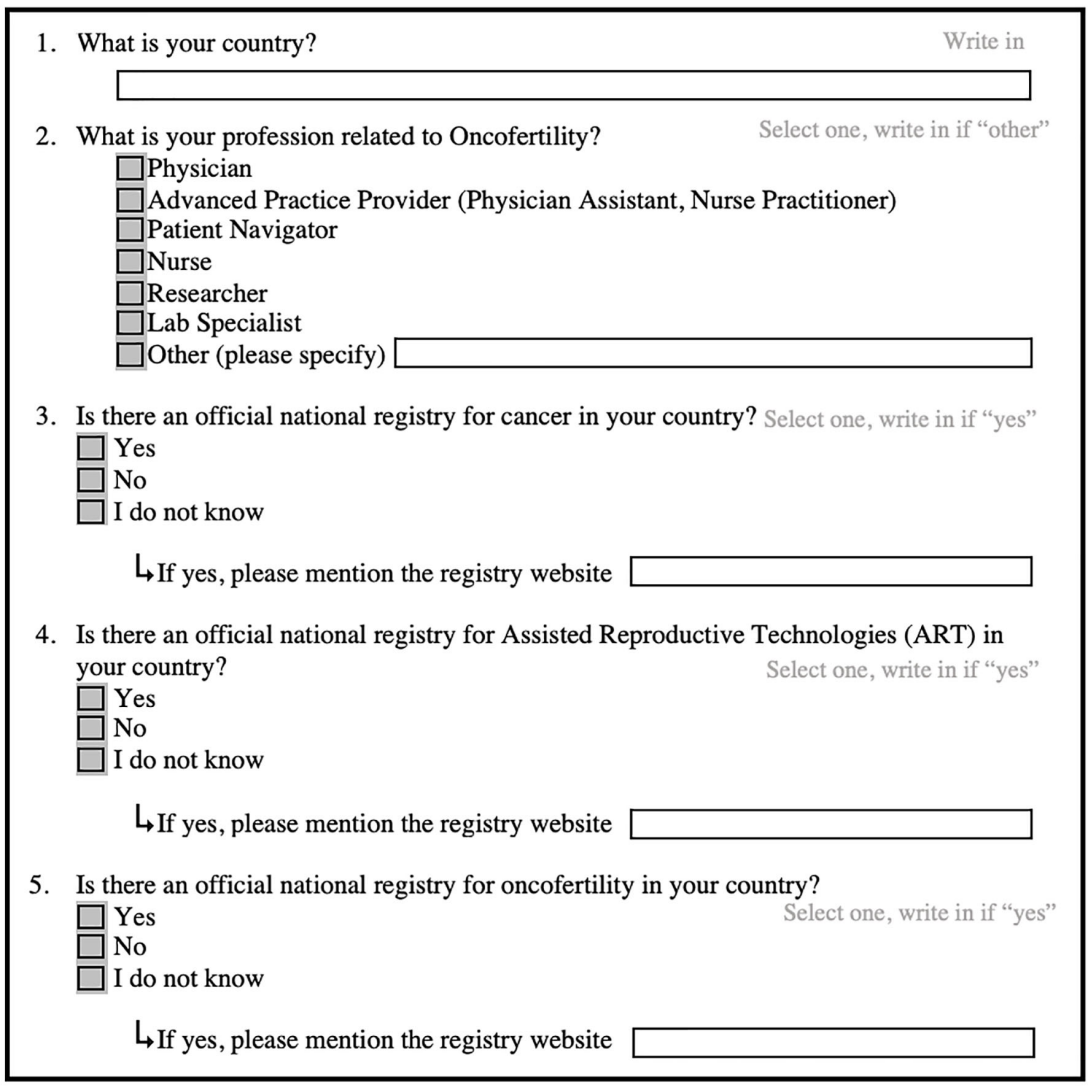
Survey Questions.

In order to identify cancer, ART and oncofertility official national registries, we conducted an online pilot survey in 2022, asking participants to report the registries available in their countries by answering the survey questions as shown in **Figure 1**.

The survey study was designed and conducted by the Oncofertility Consortium team at Michigan State University (MSU), USA. The Institutional Review Board (IRB) at MSU determined that this survey study did not constitute research that involves human subjects; therefore, additional IRB review and approval was not required.

The survey was available online on MSU Qualtrics from January 2022 to September 2022. Furthermore, a link to the online survey https://msu.co1.qualtrics.com/jfe/form/SV_brTwsVJ3vVsLqdw was shared at the 14^th^ Annual Conference of the Oncofertility Consortium, May 2-4, 2022, Pittsburgh, PA, and in the Oncofertility Consortium e-newsletters that may appeal to oncofertility care providers, such as those sent by related professional societies and academic departments.

Participation in this online survey was voluntary, anonymous and for free. Responses were confidential and no identifying information such as personal names, email addresses or IP addresses was collected. All data was stored in a password protected electronic format for scholarly purposes only.

### 2.2 Data analysis

Upon closing the survey period, we conducted a thorough internet search to authenticate each response and check whether each national registry officially exists. In the cases where survey participants from the same county reported different information about registries, all responses were reviewed to determine the accuracy of the data. Responses found to be inaccurate were discarded. After data cleaning, descriptive statistics were used to analyze the final dataset.

## 3 Results

Responses from 20 countries were collected via the online survey including Argentina, Australia, Brazil, Canada, Chile, China, Egypt, Germany, Greece, India, Japan, Kenya, Philippines, Romania, South Africa, Thailand, Tunisia, UK, USA & Uruguay. Twelve countries (60%) have official national cancer registries including Argentina, Australia, Brazil, Canada, Germany, India, Japan, South Africa, Thailand, UK, USA & Uruguay. Thirteen countries (65%) have official national ART registries including Argentina, Australia, Brazil, Canada, Germany, Greece, Japan, Romania, South Africa, Thailand, UK, USA & Uruguay. Only 3 countries (15%) have official national oncofertility registries including Australia, Germany & Japan (**Table 1**).

**Table 1:** Results of the online pilot survey.

Countries involved in the online pilot survey	Countries with an official national cancer registry	Countries with an official national ART registry	Countries with an official national oncofertility registry
N = 20 (100%)	N = 12 (60%)	N = 13 (65%)	N = 3 (15%)
1. Argentina 2. Australia 3. Brazil 4. Canada 5. Chile 6. China 7. Egypt 8. Germany 9. Greece 10. India 11. Japan 12. Kenya 13. Philippines 14. Romania 15. South Africa 16. Thailand 17. Tunisia 18. UK 19. USA 20. Uruguay	1. Argentina 2. Australia 3. Brazil 4. Canada 5. Germany 6. India 7. Japan 8. South Africa 9. Thailand 10. UK 11. USA 12. Uruguay	1. Argentina 2. Australia 3. Brazil 4. Canada 5. Germany 6. Greece 7. Japan 8. Romania 9. South Africa 10. Thailand 11. UK 12. USA 13. Uruguay	1. Australia 2. Germany 3. Japan

## 4 Discussion

According to our online pilot survey, responses from 20 countries were collected and showed that only 3 countries (Australia, Germany & Japan) have well-established official national oncofertility registries. The Australian official national oncofertility registry is part of Australasian Oncofertility Registry (AOFR) that also includes New Zealand (http://www.futurefertility.com.au/registry/). The German official national oncofertility registry is part of FertiPROTEKT Network Registry for German speaking countries that also includes Austria & Switzerland (https://fertiprotekt.com/en/patients/). The Japanese official national oncofertility registry includes Japan only and called Japan Oncofertility Registry (JOFR) (http://www.j-sfp.org/about/registry.html).

To supplement this pilot survey, we conducted a separate internet search to identify further official national oncofertility registries around the globe. Keywords were searched on both PubMed and Google alongside the countries’ names and “registries” (ex: “USA oncofertility registries”). Key terms included: oncofertility, cancer, oncology, assisted reproductive technologies, ART, IVF, sperm cryopreservation, embryo cryopreservation, oocyte cryopreservation, ovarian tissue cryopreservation, and testicular tissue cryopreservation. If providers were able to access one or more national, or multi-national registry, they were considered to have a registry available to them. Hospital level registries, local registries or regional registries were not recognized by this internet search, as we aimed to review registries available to larger populations on the national level. No additional national oncofertility registries were identified through this internet search. Therefore, the final list of countries around the globe that have official national oncofertility registries includes Australia, Austria, Germany, Japan, New Zealand, and Switzerland (**Table 2**). Some other countries such as the USA and Denmark are on their way to establish official national registries for oncofertility care. By reviewing such a global landscape, we highlight the urgent need for having a well-established official national oncofertility registry in each country to monitor oncofertility services in a way that best serves patients.

**Table 2:** Countries around the globe that have official national oncofertility registries.

Country	Official National Oncofertility Registry	Website
Australia	Australasian Oncofertility Registry (AOFR)	http://www.futurefertility.com.au/registry/
Austria	FertiPROTEKT Network Registry	https://fertiprotekt.com/en/patients/
Germany	FertiPROTEKT Network Registry	https://fertiprotekt.com/en/patients/
Japan	Japan Oncofertility Registry (JOFR)	http://www.j-sfp.org/about/registry.html
New Zealand	Australasian Oncofertility Registry (AOFR)	http://www.futurefertility.com.au/registr/
Switzerland	FertiPROTEKT Network Registry	https://fertiprotekt.com/en/patients/

Establishing a national oncofertility registry is a very challenging process. A 2019 report by the leaders of *Ferti*PROTEKT, the Oncofertility Consortium and the Danish Fertility Preservation Network discussed the logistical considerations when initiating oncofertility networks, many of which should also be considered when establishing oncofertility registries (16). In the early stages of network development, it is crucial to consider structural details such as whether the oncofertility registry will be centralized or a collaborative effort of smaller networks with shared goals and responsibilities operating under the same guidelines. Governments, institutions and cryobanks may serve as the host and primary site of the oncofertility registry, depending on the intended size and structure of the collaborative effort. Estimates of population size and local differences which could influence care logistics (insurance, cultural differences, language, etc.) should be weighed when considering the inclusion of countries or regions. FertiPROTEKT, for example, is available to all German-speaking countries (Germany, Austria & Switzerland) (16). Consideration should also be given to selecting a list of data to be collected. Current oncofertility registries often include demographics such as age and gender, as well as health information such as the type and stage of the cancer diagnosed, type of fertility preservation services attempted, as well as measures of success (e.g.: cancer and fertility preservation outcomes, quantity of samples collected for cryopreservation, pregnancy rate, and live birth rate). Among current registries, there is variation in whether members are required to fill out all information, or if some of the information is voluntary- this too is a consideration (4). Providers may be concerned about finding time to input data. Therefore, it is recommended to keep required information to a minimum and enlist the support of other professionals such as patient navigators when completing such tasks.

Startup cost can be anticipated to be an early logistical concern. Various sources of funding may be considered, including research grants, government agencies, as well as private groups and societies. Located in Australia and New Zealand, the multisite Australasian Oncofertility Registry was established by The FUTuRE Fertility research team as part of a study. The registry includes information regarding referrals, uptakes, complications, and outcomes of oncofertility services (17). The group plans to compare data to other healthcare datasets to carry out additional studies and expand the clinical picture. Collectively, this data will inform evidence-based guidelines and resources. Other considerable startup costs include cost of website creation, establishing standard operating procedures for the recruitment of centers and standardization of data collection and deposition. In 2018, the Japan Society for Fertility Preservation (JSFP) launched the Japan Oncofertility Registry (JOFR). A recent article on JOFR showed that as of January 2022, over 7000 cases from more than 100 fertility centers have been registered in Japan. JOFR aims to keep disseminating information on cancer prognoses, pregnancy rates, and other oncofertility outcomes to help monitor and improve oncofertility services in Japan (18).

A known challenge in oncofertility is its multidisciplinary nature, as success requires close coordination between reproductive medicine specialists, reproductive biologists, and oncologists in various disciplines. One report suggests that approximately 20% of patients seeking oncofertility services sought advice independently, without the recommendation of their oncologist, highlighting the need for the encouragement of collaborative care (19). When determining leadership, representation from multiple specialties may be important in forming a foundation of cooperation that is necessary for long-term sustainability.

While this paper highlights the deficit of official national oncofertility registries around the globe, some limitations should be noted. While we evaluated the availability of registries, some registries are still under development and hence excluded from our analysis. Several registries are structured differently from each other, and we are yet to understand how such variability affects the success of these registries. Utilization and adherence to registration are necessary to consider when attempting to understand the success of registries, but this data was unavailable. Challenges in obtaining this data included language barriers, lack of publicly available data, and in some cases, obstructed ability to access information when viewed from outside of the host country.

## 5 Conclusion

According to our online pilot survey, responses from 20 countries were collected and showed that only 3 countries (Australia, Germany & Japan) have well-established official national oncofertility registries. The Australian official national oncofertility registry is part of Australasian Oncofertility Registry that also includes New Zealand. The German official national oncofertility registry is part of FertiPROTEKT Network Registry for German speaking countries that also includes Austria & Switzerland. The Japanese official national oncofertility registry includes Japan only and called Japan Oncofertility Registry (JOFR). A supplementary internet search confirmed the aforementioned results. Therefore, the final list of countries around the globe that have official national oncofertility registries includes Australia, Austria, Germany, Japan, New Zealand, and Switzerland. Some other countries such as the USA and Denmark are on their way to establish official national registries for oncofertility care.

Although oncofertility services are expanding globally, very few countries have well-established official national oncofertility registries. By reviewing such a global landscape, we highlight the urgent need for having a well-established official national oncofertility registry in each country to monitor oncofertility services in a way that best serves patients. We call for the creation of such registries, with consideration to the practical challenges in doing so especially the logistical and financial challenges.

## Author contributions

All authors contributed equally in conceptualization, methodology, data collection and analysis, manuscript writing, reviewing & final editing. All authors contributed to the article and approved the submitted version.
